# A Review of the Impacts of Different Approaches for Diabetes Prevention and a Framework for Making Investment Decisions

**DOI:** 10.3390/ijerph15030522

**Published:** 2018-03-15

**Authors:** Maria L. Alva

**Affiliations:** RTI International, Washington, DC 20005, USA; malva@rti.org; Tel.: +1-202-728-1972

**Keywords:** diabetes prevention, health outcomes, economic model

## Abstract

This paper selectively reviews the economic research on individual (i.e., diabetes prevention programs and financial rewards for weight loss) and population-wide based diabetes prevention interventions (such as food taxes, nutritional labeling, and worksite wellness programs) that demonstrate a direct reduction in diabetes incidence or improvements in diabetes risk factors such as weight, glucose or glycated hemoglobin. The paper suggests a framework to guide decision makers on how to use the available evidence to determine the optimal allocation of resources across population-wide and individual-based interventions. This framework should also assist in the discussion of what parameters are needed from research to inform decision-making on what might be the optimal mix of strategies to reduce diabetes prevalence.

## 1. Introduction

High risk of diabetes imposes high health risks and economic burden. Progression from impaired glucose to type 2 diabetes increases the risks of CVD and other diabetes-related complications which leads to high medical costs, and other economic losses (e.g., absenteeism and presentism in the labor force).

According to the International Diabetes Federation [1], diabetes currently affects at least 415 million people worldwide (this figure might be higher as many individuals are undiagnosed). This number is estimated to raise. The regions with the highest prevalence (9% and above), are the Middle East and North Africa, South and Central America, and the Western Pacific. The highest number of people with diabetes are found in the U.S. (29.3 million), India (69.2 million), and China (109.6 million). While in developed countries, most people with diabetes are above the age of retirement, in developing countries, those most frequently affected are in what should be the most productive years of their lives, between 35 and 64 years old.

There is not a single dominant cause of type 2 diabetes risk—while a variety of factors such demographic characteristics and clinical factors have been shown to contribute to a high likelihood of diabetes, obesity is the principal risk factors associated with type 2 diabetes, irrespective of age [2]. The two main corollaries to the caveat that this association does not imply causation are that not all obese individuals develop diabetes (genes might play a role too), and that fat, and specifically visceral fat, might be better predictors than weight [3]. Focusing on curbing obesity to prevent diabetes is a leading strategy both because obesity is a modifiable factor and because several clinical trials and observational studies show that 5–7% weight loss is sufficient to prevent individuals with impaired glucose tolerance developing type-2 diabetes [4,5]. To the extent that diabetes is preventable, diabetes related complications such as macrovascular and microvascular complications along with their direct (healthcare expenditure and years of life lost) and indirect costs (presentism and absenteeism in the labor market), can be avoided.

The goal of this paper is twofold: to review the impacts of different individual-level and population wide approaches for diabetes prevention and to present a framework for decision-making. The goal of the selective review is to look at the evidence for feasibility and effectiveness of interventions designed to prevent diabetes and the extent to which effects are replicable across population with different demographic characteristics and risk profiles. The growing number of evaluations of diabetes prevention programs (DPP) have led to a few systematic reviews [6,7] showing that intensive lifestyle interventions are successful at generating clinically meaningful weight loss. There are however at least four caveats to taking at face value the available evidence. First, because most studies take place in developed countries, it is important to see to what extent the effects of lifestyle interventions are replicable in other contexts such as those of low- and middle-income countries. Second, we need studies that quantify and compare through mixed indirect comparisons, whenever direct ones are missing, the overall effects of the most common, but vastly different, types of prevention interventions such as lifestyle, pharmacological, and bariatric surgery. Direct evidence on pair-wise comparisons alone cannot determine which of several treatments is the most effective or cost-effective. Indirect comparisons of A versus B and A versus C should be used to infer the evidence of B relative to C when that direct evidence is lacking [8]. Third, most of the evidence available lacks long term follow up. Important to know is the extent of mean reversion in outcomes. Even in studies with long term follow up, accounting for survivorship bias is often missing. Fourth population based approaches, while theoretically convincing, do not have much in the way of empirical evidence on diabetes related incidence. For example, there is enough evidence to show that sugary drinks are a major contributor to the obesity epidemic [9] yet the evidence on SSBs (sugary sweetened beverages) taxes on diabetes incidence is not available.

Because of these caveats and gaps in the literature, it is important to outline a framework that highlights the type of parameters that are important for cost-effective decision making and that can guide us through the best allocation of public resources by championing effective policies over poor ones. This paper illustrates a hypothetical transition model of diabetes risk (low and high) where the goal is minimizing diabetes prevalence in the population subject to a budget constraint. The proposed framework would be useful to decision-makers to allocate resources between different types of interventions with different costs and efficacy rates. For example, the framework could be useful to estimate the monetary cost needed to reach a predetermined national goal, such as preventing 30% of new cases, and the amount of funding to be allocated to different programs to achieve that goal optimally.

Broadly, individual approaches focus on high-risk individuals through direct interventions. Because individual level interventions are demand driven, success often depends on the commitment and adherence to the program by participants. Population level interventions are supply driven. They change the economics (tax on sugar), the physical landscape (e.g., pedestrian walkways and/or bike paths), and the regulatory environment (e.g., changing the fat, salt and/or sugar content in foods), which then should nudge individuals toward good choices without requiring active commitments, unless opting in is needed (e.g., a tax on sugar-sweetened beverages still requires individuals to choose not to purchase SSBs and bike paths still require individuals to choose to use them). Comparing the efficacy of individual and population level strategies for diabetes prevention is important for budget allocation and to strengthen efforts to slow the rate of increase of diabetes prevalence. This is particularly important for lower-income countries facing rapid increases in diabetes incidence while also having low health spending budgets, a dearth of health workforce, access to health information and other preparedness measures [10].

The paper is structured as follows. Section 2 discusses evidence based on diabetes incidence or improvements in diabetes risk factors such as weight, glucose or glycated haemoglobin (HbA1c) resulting from individual (i.e., diabetes prevention programs, bariatric surgery and pharmacological interventions) and population-wide based diabetes prevention interventions such as food taxes. Section 3 discusses an economic model to minimize diabetes incidence subject to a budget constraint. Section 4 offers a possible research agenda on diabetes preventions, emphasizing the greatest needs to decision makers.

## 2. Literature Review

### 2.1. Selection Criteria

Given the size of the literature, this review started by necessity as selective. A literature search in PubMed to identify relevant articles published in English between January 2000 and October 2017 using the following key terms to denote well known individual and population level interventions (diabetes prevention program or weight loss intervention or bariatric surgery or worksite physical activity or joint use agreements or public transportation systems or SSB taxation or nutritional labeling) and type two diabetes returns more than 15,000 articles. However, when restricting this search by adding diabetes incidence in all fields we have only 47 studies. Out of this small selection, not all studies reported as an outcome diabetes incidence (i.e., direct reduction); deterministic markers of diabetes progression such as glucose or HbA1c levels; or improvement in the leading diabetes risk factor (e.g., BMI/weight). Some studies even lacked statistical significance tests (*p*-values) alongside the analyses performed. One of the reasons interventions geared to stave off diabetes incidence do not report this outcome might be that most studies lack the funding to have both a large sample size and the time duration needed to monitor the disease progression. The strategy in this paper was then to look at systematic reviews and seminal papers on individual level interventions and population-wide interventions that explicitly looked at diabetes incidence and from these conduct snowballing searches.

### 2.2. Individual Level Interventions

#### 2.2.1. The Diabetes Prevention Program

Perhaps the most well-known and certainly the most published individual-based approach is the Diabetes Prevention Program (DPP) where participants attend weekly classes covering nutrition, exercise and motivation. The goal of the DPP is for participants to lose at least 5% (in most trials the goal was 7%) of their body weight and increase physical activity to 150 min a week [11,12]. The rationale for the goal is based on existing evidence showing the link between weight loss and diabetes incidence [13,14].

The DPP has been translated into programs delivered by different healthcare providers and instructors (e.g., primary care practitioners, pharmacists, dieticians, fitness instructors and community health workers) and in different settings (hospitals, gyms, community centers, and online). The program is based on the replication of randomized trials such as the Diabetes Prevention Program in the US [15], the Finland Diabetes Program [16,17,18], and the Da Qin Study in China [19,20]. These programs are targeted to adults with either impaired fasting glucose (IFG) (fasting plasma glucose of 100–124 mg/dL) or impaired glucose tolerance (IGT) (2-h post-load glucose of 141–199 mg/dL) or both. Lifestyle intervention participants in the DPP trial had a 58% reduction in the incidence rate of diabetes and 31% in the metformin treated group over 2.8 years, compared with placebo. Diabetes incidence in the 10 years since DPP randomization was 34% (CI: 24–42) in the lifestyle group and 18% (CI: 7–28) in the metformin group compared with placebo, showing that prevention or delay of diabetes with lifestyle intervention or metformin can persist in the medium to long run. The DPP is designed to last four months, with maintenance sessions happening through the remaining of the year and it is worth noting that the follow up design of that study included an effort to continue offering the lifestyle program. The Finnish study had reported similar numbers a year earlier (i.e., risk reduction of 58% in 3.2 years): the average proportion of subjects in whom impaired glucose tolerance progressed to diabetes was 3% per year in the intervention group and 6% per year in the control group. The seven-year follow up risk reduction was 43%. In the Da Qin study, the average annual incidence of diabetes was 7% for intervention participants versus 11% in control participants, with 20-year cumulative incidence of 80% in the intervention groups and 93% in the control group [19]. The long-term results are the product of the fact that the cumulative incidence is capped at 100% and, as time progresses, there are fewer people at risk for incidence. There is also unaccounted survivorship bias over time, as individuals who delayed the onset of diabetes live longer than those in the control group and therefore increase their risk of having diabetes. Completion rates in these trials were large, for example 96% of lifestyle interventions finished the DPP curriculum. There is evidence from translational studies that completing the program allows individuals to attain the 5–7% medically relevant weight loss target [21]. However, in half of all studies conducted thereafter, completion rates were ≤10% [6]. Finding ways to engage individuals for the entire program is challenging.

#### 2.2.2. Bariatric Surgery

Weight loss is hard to maintain, among other things because it reduces a person’s metabolic rate [22]. A growing popular procedure, among individuals that are relatively young (typically below the age of 60) and with a body mass index ≥30, is bariatric surgery. There are two basic types of bariatric surgery: the Roux-en-Y gastric bypass (RYGB), and the laparoscopic adjustable gastric banding (LAGB). Numerous observational studies and few randomized controlled trials have compared bariatric surgery with non-surgical treatment for obesity. The only study that to our knowledge looked at whether bariatric surgery could be an effective instrument to prevent diabetes is the Swedish Obese Subjects (SOS) study [23]. While the biggest limitation of that study is that it was not a randomized study, the authors followed participants over 15 years and found that the bariatric-surgery group incidence rate of diabetes was of 6.8 (95% CI, 5.7–8.3) per 1000 person-years while the control group was of 28.4 cases (95% CI, 25.7–31.3) per 1000 person-years.

Gloy et al. [24] meta-analyzed bariatric surgery with non-surgical treatment for obesity on different health outcomes. In that review, there are a handful of studies that either combined individuals with diabetes and at risk of diabetes or studies with only individuals with type 2 diabetes at baseline, with different definitions for diabetes remission, showing bariatric surgery to be significantly more effective than lifestyle intervention in inducing weight loss and remission of type 2 diabetes and metabolic syndrome. For example, the studies (five, including only individuals with type 2 diabetes) reported mean difference −1.5% ((ranging from −1.9 to −1.1), *p* < 0.001) in HbA1c more after bariatric surgery than after non-surgical treatment. Among obese participants with T2DM, bariatric surgery with two years of an adjunctive low-level lifestyle intervention resulted in more disease remission than did lifestyle intervention alone. Holter et al. (2016) found that glucose control is initially superior following Roux-en-Y gastric bypass (RYGB), as compared with laparoscopic adjustable gastric banding (LAGB), but the effect is not sustained beyond 10% weight loss [25]. There are few follow up studies available. Courcoulas et al. (2015) followed individuals over three years [26] and showed that use of diabetes medications was reduced more in the surgical groups than the lifestyle intervention-alone group, and that none of the intensive lifestyle intervention participants went from using insulin or oral medication at baseline to no medication at Year 3 (*p*  < 0.001). Mean (SE) reductions in percentage of body weight at years were the greatest after RYGB at 25.0% (2.0%), followed by LAGB at 15.0% (2.0%) and lifestyle treatment at 5.7% (2.4%) (*p* < 0.01).

#### 2.2.3. Pharmacological Interventions

There are two forms of pharmacological intervention for diabetes prevention: oral diabetes drugs, of which the most commonly studied are Acarbose and Metformin, and anti-obesity drugs. While meta-analyses have shown a significant benefit of pharmacological intervention compared with controls (hazard ratios 0.70 for oral diabetes drugs and 0.44 for anti-obesity drugs) [27], the set of comparators is highly heterogeneous. There are few useful head to head comparisons. For example, in the DPP, metformin was much less effective than the lifestyle intervention in delaying the onset of diabetes (31% vs. 58%). In the Study to Prevent Non-Insulin-Dependent Diabetes Mellitus known as the STOP-NIDDM trial [28], acarbose reduced the risk by 32%, similar to what was found for metformin in the DPP trial but, because the study participants are very different between these studies, no inferences can be made between acarbose and lifestyle interventions. There are three additional limitations with pharmacological interventions: (1) the long run incidence of diabetes after discontinuation of drugs is unknown; (2) drugs have undesirable side effects and are contraindicated for some individuals; and (3) drugs require regular monitoring, which adds to the cost of managing the disease.

### 2.3. Population Level Interventions

#### Sugar-Sweetened Beverages (SSB) Taxes

The World Health Organization (WHO) has proposed using price policies to promote healthier diets [29]. Taxes on SSBs have been enacted in over 30 countries. Based on a meta-analysis of 17 cohorts in the United States and the UK, Imamura et al. [30] found that an additional 1 serving/day of SSBs was associated with an increased incidence in T2D by 18% (95% CI: 9%, 28%), or by 13% (6%, 21%) adjusting for adiposity over 10 years. This reduction is comparable to an estimated reduction in T2D incidence from a microsimulation model [31] of 1.8–3.4% associated with a 20% decrease in soda consumption over 10 years. The American Heart Association recently released a scientific statement recommending reductions in added-sugar intake to no more than 100–150 kcal/day [32] while citing SSBs as the primary source of added sugars in the American diet [33]. SSBs contribute to weight gain because of their high caloric content and low satiety [34,35].

Studies of soda taxes have shown they lead to lower sales of sugar-sweetened beverages [36], for example, Falbe et al. [37] studied the effect of a $0.01/oz. tax on SSBs implemented in Berkeley, CA in 2015. Four months after implementation, SSB consumption had decreased by 21%, while it had increased by 4% in comparison cities over the same period. In Mexico, Colchero et al. (2017) [36] found that a one peso per liter tax resulted in absolute and relative widening of the differences between the post-tax volume and its counterfactual over one year post-tax of −11 mL/capita/day (−5.6% relative to the counterfactual). The decrease in purchases of non-carbonated sugar sweetened beverages was much larger than for sodas. Relative to the counter factual, the authors showed that reductions were higher among the households of low socioeconomic status (9% decline on average). This is potentially important because taxes are a transfer from the pockets of soda buyers to those people whose health might be improved by the policy. Those who are still buyers, because they are addicted, have then less money for other goods. Important empirical questions are then: in which population are health benefits largest? In those highly at risk or in those with low risk? How sensitive are individuals in lower socioeconomic groups to taxes? This is especially important in countries where income inequality is high. Do elasticities of demand vary across the income distribution? Without delving into redistribution matters, taxes appear to be the single best buy: the marginal cost of the policy is close zero while increasing government revenue.

Because taxes are levied on beverage distributors, it is important to know how much of the tax is passed through to consumers as higher retail prices vs being paid by distributors or manufacturers [38]. Exploiting the fact that the Philadelphia International Airport straddles two cities and that the city of Philadelphia implemented, on 1 January 2017, a tax of 1.5 cents per ounce on SSBs, Cawley et al. (2017) estimated the extent to which stores in the Philadelphia side of the airport raised retail prices compared to stores located on the untaxed adjacent city of Tinicum. The pass-through of the tax on SSBs in the Philadelphia airport (93%) was higher than that found in Berkeley, California, which studies estimated was in the range of 43–69% [39,40].

There are at least three unknowns s in this line of research. First, there is no direct proof of how effective soda taxes are in reducing diabetes incidence. We have evidence on the indirect components: we know that consumption of SSBs is highly linked to diabetes incidence and we have evidence, albeit small, of taxes curbing consumption. SSBs taxes to have the intended consequences require consumers to make choices. Given possible heterogeneity in response, it is unclear whether taxes will induce the desired behavior among individuals at risk of diabetes. Producers’ regulation, such that no single beverage should contain more than x percentage of the recommended daily sugar intake, might be a better policy option. Second, is the relationship between reduction in consumption and reduction in T2D incidence linear? It might not be qualitatively equivalent to drink five glasses a day and decrease consumption by one than it might be to drink two glasses and cut consumption to one. Third, taxes on SSBs thus far have been highly localized and we are yet unaware of spillover effects, i.e., does demand for SSBs increase in adjacent areas? A point made by several authors, which is both obvious and important, is that taxes should be enacted at high levels of geographic aggregation to make them harder to evade.

Other potentially promising population-wide interventions for which we do not yet have evidence on the endpoints of interest are: the role of the environment (e.g., walkable/bikable environments and the availability of public transportation); menu labeling; and nutrition education interventions [41].

## 3. An Economic Framework

Several interventions can be applied to prevent type 2 diabetes with varying costs, health effects and targets (general population versus population at risk). An economic framework that accounts for budget constraints and efficacy can allow us to optimize prevention at minimum cost. Imagine that time for each new generation is divided into three periods and the same pattern is repeated across cohorts. We can normalize the population to one and assume that a fraction of the initial population at baseline has either already developed diabetes (D); has high risk ($S_{h}$); or has low risk of developing diabetes $\left(S_{l}\right)$. For simplicity, let us also assume that that, once a person has become high risk or developed diabetes, he/she does not revert to not having the condition (this is a simplification since studies have shown that people can achieve normoglycemia after a diabetes diagnosis). The goal of a decision maker would be to minimize the incidence of diabetes across time subject to a budget constraint.

The transition matrix, T, of this set up looks as follows:

(1) $$\left[\begin{array}{ccc} D\overset{1}{\to }D & D\overset{0}{\to }S_{h} & D\overset{0}{\to }S_{l}\\ S_{h}\overset{p_{hd}}{\to }D & S_{h}\overset{1-p_{hd}}{\to }S_{h} & S_{h}\overset{0}{\to }S_{l}\\ S_{l}\overset{0}{\to }D & S_{l}\overset{p_{lh}}{\to }S_{h} & S_{l}\overset{1-p_{lh}}{\to }S_{l} \end{array}\right]$$

where

$p_{hd}$ = probability of being high risk and transitioning to having diabetes from one period to the next;
$p_{hh}$ = $1-p_{hd}$ = probability of being high risk and remaining high risk from one period to the next;
$p_{lh}$ = probability of being low risk and transitioning to high risk from one period to the next; and
$p_{ll}$ = $1-p_{lh}$ = probability of being low risk and remaining low risk from one period to the next.

Note that because all rows in T need to sum to one (states are exhaustive), the matrix has only two parameters of interest: $p_{hd}$ and $p_{lh}$.

For simplicity, assume there are only two types of policies: individual level interventions and population wide interventions. Individual-level interventions are designed to specifically target high risk individuals and population-wide interventions only prevent low-risk individuals from becoming high risk. The decision maker’s choice is how much to spend on prevention, where $C_{l}$ is the cost of population-wide interventions and $C_{h}$ is the costs of individual-level interventions. If no resources are allocated to prevention, both $C_{l}$ and $C_{h}$ are equal to zero, and transitions to worse off states happen with certainty at a rate equal to the baseline transition parameters $\pi_{hd}$ and $\pi_{lh}$ (incidence rate without interventions) Probabilities $p_{hd}$ and $p_{lh}\;$ are a function of how much is spent on prevention:

(2) $$p_{hd}=f\left(C_{h}\right)=\pi_{hd}\;e^{-\beta_{h}C_{h}}$$

(3) $$p_{lh}=f\left(C_{l}\right)=\pi_{lh}\;e^{-\beta_{l}C_{l}}$$

where $\beta_{h}$ and $\beta_{l}$ are parameters (not always) found in the literature and denote how effective individual and population-wide interventions are. These represent the return to the marginal dollar spent on the program. The convexity assumption in the functional form chosen for $p_{hd}$ and $p_{lh}$ illustrates that, as transition probabilities to worse off states fall from $\pi_{hd}$ and $\pi_{lh}$, costs increases slowly at first, then rapidly and finally slowly again. This assumption also implies that $f^{\prime \prime }\left(C\right)>0$. $f^{\prime }\left(C\right)=-\beta p$ has an appealing interpretation: the percentage change in the probability of transitioning to a worse off state, p, that results in the additional expenditure in the targeted intervention diminishes at a rate equal to the effect of the intervention itself (i.e., β).

The budget constraint is as follows:

(4) $$B=C_{D}+C_{h}+C_{l}$$

where *B* is the amount the country spends on prevention efforts. The cost of treating diabetes $C_{D}$ can be considered as fixed for illustrative purposes. Without loss of generality, we can then assume that $C_{h}+C_{l}=1$.

At Period 1, we start with an initial vector, $v_{1},$ $\begin{array}{ccc} D, & S_{h}, & S_{l} \end{array}$. Developed and developing countries might have different risk profiles in terms of the initial population distribution across high risk and low risk groups. Those initial differences in the distribution of the baseline population can lead to different optimal choices of policy mixes. 

Period 2 vector is the result of multiplying $v_{1}$ by the transition matrix in Equation (1):

(5) $$\begin{array}{ccc} D+p_{hd}S_{h} & (1-p_{hd})S_{h}+p_{lh}S_{l} & (1-p_{lh})S_{l} \end{array}$$

The result is another 1 × 3 vector $v_{2}.$ Using the same process, i.e., multiplying $v_{2}$ by the transition matrix, Period 3 vector is:

(6) $$D+p_{hd}S_{h}+p_{hd}\left((1-p_{hd}\right)S_{h}+p_{lh}S_{l})\;\;(1-p_{hd})(1-p_{hd})S_{h}\;\;+\;p_{lh}S_{l}+p_{lh}(1-p_{lh})S_{l}{\left(1-p_{lh}\right)}^{2}S_{l}^{2}$$

Because our goal is to minimize diabetes incidence in Periods 2 and 3, our objective function, after substituting Equations (2) and (3) and re-arranging terms, is:

(7) $$obj=2D+\pi_{hd}\;e^{-\beta_{h}C_{h}}\left[\left(3-\pi_{hd}\;e^{-\beta_{h}C_{h}}\right)S_{h}+\pi_{lh}S_{l}\;e^{-\beta_{l}C_{l}}\right]$$

The initial vector of states and the amount spent on prevention (the controls) determine the overall prevalence. The Lagrangian function is:

(8) $$L=obj+\lambda \left(1-C_{h}-C_{l}\right)$$

The level of spending $C_{h}^{*}$ and $C_{l}^{*}$ that minimizes diabetes prevalence in Periods 2 and 3 are obtained by taking the first derivative of L with respect of $C_{h}$ and $C_{l}$. While there are no close form solutions (costs remain as terms in the derivative), the resulting comparative statics are intuitive:

$\frac{\partial C_{h}^{*}}{\partial \beta_{h}}>0;\;\frac{\partial C_{l}^{*}}{\partial \beta_{l}}>0$, meaning that spending increases with the level of effectiveness.
$\frac{\partial C_{h}^{*}}{\partial S_{h}}>0;\;\frac{\partial C_{l}^{*}}{\partial S_{l}}>0$, meaning that the more high or low types there are, the more one would want to spend locally, within those cohorts. For example, countries with a large fraction of the population at high risk $S_{h}$, at the margin should spend on $C_{h}$.
$\frac{\partial C_{h}^{*}}{\partial S_{l}}<0;\;\frac{\partial C_{l}^{*}}{\partial S_{h}}<0$ is true as expenditures on high and low cohorts comes from the same budgets.

Note that, to avoid parameter overload, $C_{h}$ is assumed to convey the unit cost times the proportion of the population that gets the intervention, while we can assume that $C_{l}$ covers 100% of the population.

## 4. Discussion

Decision-making should be based on parameters derived from randomized controlled trials (RCTs) or best available evidence from meta-analyses. Currently, many parameters are missing from the literature and specifically evidence on population wide interventions. Most research has been conducted on individual-level interventions for diabetes prevention. Despite the data gaps, or because of them, there is consistent evidence that individual-based interventions are effective across a range of settings and modes of delivery. Population-based approaches have instead more varied and modest effects and not much in the way of evidence on diabetes-related outcomes. Conditional on availability of estimates, emphasis should be placed on larger studies of longer duration that are better powered to detect changes in diabetes incidence resulting from individual-level and population-wide interventions. At a minimum, to solve the simple model illustrated in Section 3 and know how much it would be optimal to spend for individual versus population level-interventions, we need information on:

- diabetes prevalence;
- estimates of the fraction of the population at high and low risk;
- baseline probability risk of transitioning to worse off states in the absence of interventions;
- cost estimates of providing individual and population preventive interventions; and
- the cost-effectiveness of these in preventing or delaying diabetes (the betas of the economic framework).

Note that the model did not need to have two types of interventions as these are not modeled as complements or substitutes but rather independent of each other. Given the lack of evidence on the effect of SSBs taxes on diabetes incidence; their effects across different levels of risks and the small effects of taxes on consumption, it seemed reasonable to assume that a population-wide intervention such as that would have an impact on low risk individuals only.

There are four important questions around the use and interpretation of betas in the model which require additional information and further model refinements:

- *To what extent can we assume that programs are replicable and scalable?* We have some evidence that individual-level interventions are not always scalable as translational studies, rolled out under real world conditions, often do not manage to replicate results from RCTs conducted under more controlled conditions. Others have posed this question in terms of what differences may exist between efficacy (the effect of the intervention under ideal and controlled conditions) and effectiveness (performance under “real-world” conditions) and, more narrowly, on how well users adhere to recommendations [42]. In the DPP trial, for example, persons already taking drugs that lower glucose, persons with serious medical conditions, and persons unable to adhere to the intervention during the run-in period were excluded, therefore the results of the trial should not be expected to necessarily apply to the average person at risk of diabetes. We also know from the DPP translational studies that adherence is harder among younger, non-Caucasian (race might, however, be a catch all variable) individuals with lower socio-economic status (based on education and income).
- *To what extent are there complementarities between individual level and population wide interventions?* There is currently no quantifiable evidence on the interaction between individual level interventions and different levels of population wide interventions and their impacts on diabetes incidence. Joint-use agreements, improved public transportation systems, and nutritional labeling are some examples of population-wide interventions that may make it easier for those enrolled in individual-level interventions like the DPP to succeed by giving people access to facilities for physical activity, increase physical activity automatically, and by helping participants to adhere to nutritional guidelines.
- *To what extent effects are a function of the number of people enrolled?* For example, one dollar spread over more people should generate less value. This might not be the case for population level interventions, $\beta_{l}$. This adds additional complexity to the model and changes the comparative statics previously discussed: if $\beta_{h}$ decreases with the size of the population, there arrives a point where the optimal choice is to start investing in population-wide interventions even if $S_{h}$ is high.
- *To what extent can we assume that individuals respond similarly to the same intervention?* It is thus unlikely that a program would have the same risk reduction on all individuals. For example, in the DPP trial metformin was most effective in people 25–44 years old and in those with a body mass index of 35 and had no significant effect among older individuals [43]. For optimal resource allocation, we need to know the policy impact on each risk group because if individuals with access to a treatment are not the most likely to benefit from it, rolling out the program to them decreases its average effectiveness while rolling the program more broadly might increase effectiveness. Knowledge of heterogeneity can help us assign different treatments to individuals and to balance competing objectives, such as reducing cost, maximizing average outcomes, and reducing variance in outcomes within a given population [44].

Having a framework can help stakeholders ask more and better questions. A model can yield important insights on how budgets should be allocated to programs and how programs should be best offered to different people.
